# Identification and evaluation of UL36 protein from *Dermacentor silvarum* salivary gland and its interaction with *Anaplasma ovis* VirB10

**DOI:** 10.1186/s13071-020-3975-9

**Published:** 2020-02-27

**Authors:** Muhammad Uzair Mukhtar, Naveed Iqbal, Jifei Yang, Qingli Niu, Shuaiyang Zhao, Zhi Li, Yaru Zhao, Muhammad Rashid, Ze Chen, Guiquan Guan, Zhijie Liu, Hong Yin

**Affiliations:** 1 State Key Laboratory of Veterinary Etiological Biology, Key Laboratory of Veterinary Parasitology of Gansu Province, Lanzhou Veterinary Research Institute, Chinese Academy of Agricultural Sciences, Xujiaping 1, Lanzhou, 730046 Gansu People’s Republic of China; 2 Jiangsu Co-innovation Center for Prevention and Control of Important Animal Infectious Diseases and Zoonoses, Yangzhou, 225009 People’s Republic of China

**Keywords:** *Anaplasma ovis*, *Dermacentor silvarum*, Salivary gland, Yeast two-hybrid, GST pull down, Protein-protein interaction

## Abstract

**Background:**

*Anaplasma ovis* is a gram-negative, tick-borne obligate intraerythrocytic pathogen, which causes ovine anaplasmosis in small ruminants worldwide. VirB10 of *A. ovis* is an integral component of the Type IV Secretion System (T4SS). The T4SS is used by bacteria to transfer DNA and/or proteins undeviatingly into the host cell to increase their virulence. To more thoroughly understand the interaction between *A. ovis* and *Dermacentor silvarum*, a vector containing the *virb10* gene of *A. ovis* was used as a bait plasmid to screen interacting proteins from the cDNA library of the *D. silvarum* salivary gland using the yeast two-hybrid system.

**Methods:**

The cDNA of the *D. silvarum* salivary gland was cloned into the pGADT7-*Sma*I vector (prey plasmid) to construct the yeast two-hybrid cDNA library. The *virb10* gene was cloned into the pGBKT7 vector to generate a bait plasmid. Any gene auto-activation or toxicity effects in the yeast strain Y2HGold were excluded. The screening was performed by combining the bait and prey plasmids in yeast strains to identify positive preys. The positive preys were then sequenced, and the obtained sequences were subjected to further analyses using Gene Ontology, UniProt, SMART, and STRING. Additionally, the interaction between the bait and the prey was evaluated using the glutathione S-transferase (GST) pull-down assay.

**Results:**

A total of two clones were obtained from the cDNA library using the yeast two-hybrid system, and the sequence analysis showed that both clones encoded the same large tegument protein, UL36. Furthermore, the proteins GST-UL36 and His-VirB10 were successfully expressed *in vitro* and the interaction between the two proteins was successfully demonstrated by the GST pull-down assay.

**Conclusions:**

To our knowledge, this study is the first to screen for *D. silvarum* salivary gland proteins that interact with *A. ovis* VirB10. The resulting candidate, UL36, is a multi-functional protein. Further investigations into the functionality of UL36 should be carried out, which might help in identifying novel prevention and treatment strategies for *A. ovis* infection. The present study provides a base for exploring and further understanding the interactions between *A. ovis* and *D. silvarum*. 
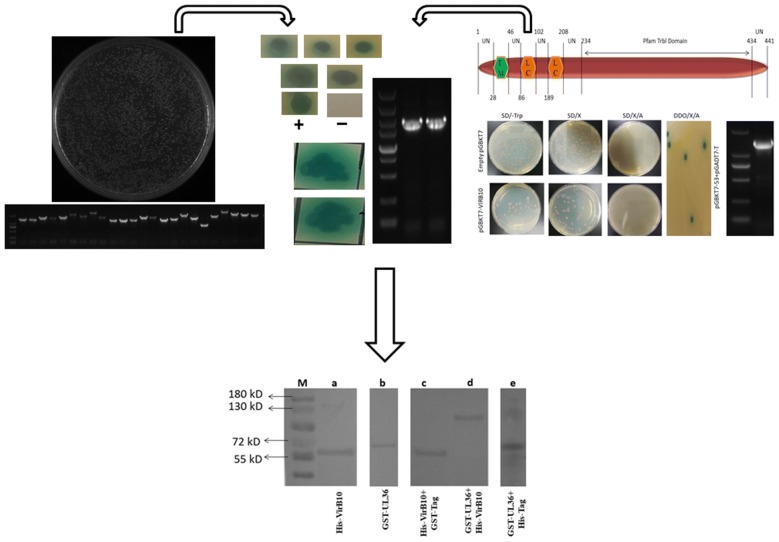

## Background

*Anaplasma ovis* is a gram-negative, tick-borne obligate intraerythrocytic pathogen [1], which causes ovine anaplasmosis in small ruminants worldwide [2]. The symptoms of the disease include severe anemia, fever, weight loss, spontaneous abortion, jaundice, and even death, in sheep and goats [3]. The economic impact of *A. ovis* is becoming increasingly serious in countries where sheep and goats are the most important livestock [4]. The transmission of this pathogen is primarily *via* tick bites [5]; the tick vector species are mainly species of the genera *Dermacentor*, *Rhipicephalus* and *Hyalomma* and play a crucial role in maintaining and spreading the pathogen [5–7]. As yet, the vertical transmission of *A. ovis* in ticks has not been substantiated. It is known that *A. ovis* initially infects the midguts of ticks, and then migrates to the salivary glands where the pathogens are transmitted to the new hosts during blood-feeding [8, 9].

Bacteria use the Type IV Secretion System (T4SS) to undeviatingly transfer virulence, DNAs and/or proteins, into the host cell [10]. The T4SS is a 1.1 MDa multi-protein complex, composed of 12 proteins (including VirB1-11 and VirD4), and this protein complex resides within the membrane bilayers of bacteria [11]. The core complex of T4SS comprisesVirB10, in conjunction with other proteins [12]; therefore, VirB10 is an integral component of the system [12]. The core complex is embedded in the outer and inner membranes and plays an active role in the transfer of the Type IV system’s substrate [13].

A previous study has shown that VirB10 is homologous to TrbI and *vir* gene proteins, which mediate conjugation-like processes [14]. Comparative studies of the T4SS of unrelated bacteria revealed that the VirB7-VirB11 proteins, including VirB10, are the basic component of the T4SS [15]. A study of the *Agrobacterium tumefaciens* secretion system found that VirB8-VirB10 interact with each other and play protein initiator roles during the assembly process [16, 17]. VirB10 is an essential component of a trans-membrane pathway for the exportation, biosynthesis and transmission of DNA. The T4SS of obligate intracellular pathogens have a preeminent role in survival, nutrition and virulence [18]. It has been demonstrated that the T4SS also functions in the tick-infection stage of *Anaplasma* and *Ehrlichia* life-cycles [19, 20]. As ascertained previously, T4SS is associated with a bacterial conjugal system that transports effector macromolecules produced by bacteria into eukaryotic target cells [21]. The regulation and expression of T4SS protein genes, including *virb10*, were investigated, and replication, cellular transportation, and intracellular survival were explored as potential functions of T4SS in rickettsial pathogens [22].

There have been previous studies involving the screening and identification of possible host or proteins of bacterial origin that interact with *Anaplasma* spp. (other than *A. ovis*), but few interacting proteins from ticks have been reported. In this study, to more thoroughly understand the interaction between *A. ovis* and *D. silvarum*, a recombinant *virb10* gene was used as bait to screen potential interacting proteins from the cDNA library of the *D. silvarum* salivary gland using a yeast two-hybrid system.

## Methods

### Isolation of the tick salivary glands

To collect salivary glands, *A. ovis* infection-free adult ticks (*D. silvarum*) were used. The ticks were maintained for several consecutive generations at the State Key Laboratory of Veterinary Etiological Biology of Lanzhou Veterinary Research Institute, China, under optimum temperature and humidity. Ticks were tested for *Anaplasma* infection by PCR using the primer pairs EE1/E2, AB1f/1r, SSAP2f/2r, AmargMSP4Fw/4Rev and MSP45/43 [23–26]. To collect the salivary glands, each tick was dissected into two parts between coxa 1 and coxa 2 using a microtome. Then, the anterior part was removed from the posterior using sterile tweezers. The salivary glands were carefully detached from the anterior part without damaging the midgut. The salivary glands were then collected into a sterile 1.5 ml tube containing 500 μl Trizol (Sigma-Aldrich, Cleveland, OH, USA). All dissection steps were performed with great care under a stereomicroscope to avoid contamination by the midgut fluid.

### Construction of a yeast two-hybrid cDNA library of salivary glands

The salivary glands collected from *D. silvarum* were sent to Takara (Dalian, China) for the construction of a yeast two-hybrid cDNA library. The total RNA of the salivary glands was extracted and reverse transcribed into first-strand cDNA. Following normalization treatment using a cDNA Normalization Kit (Invitrogen, Carlsbad, CA, USA) and short fragment removal using Make Your Own “Mate & Plate™” Library System (Clontech, Mountain View, CA, USA), the salivary gland cDNA was cloned into the pGADT7-*Sma*I vector (prey plasmid) for the construction of the cDNA library.

### Bait plasmid construction

For the construction of the bait plasmid (*A. ovis virb10*), the genomic DNA was extracted from 200 μl *A. ovis* (Heibei strain)-infected whole blood using the QIAamp DNA Mini Kit (Qiagen, Hilden, Germany) according to the manufacturer’s instructions. The *virb10* gene fragment was amplified by PCR from the genomic DNA, based on the specific primers VB10otF/R (Table 1). Extracted DNA (2 μl) was used as the template in a 25 μl volume PCR reaction mixture, comprising 12.5 μl Takara PCR Master Mix, 1 μl forward and reverse primers, and nuclease-free water. PCR conditions consisted of initialization at 94 °C for 4 min, followed by 35 cycles of denaturation at 94 °C for 30 s, annealing at 58 °C for 30 s, extension at 72 °C for 1 min 30 s, and a final extension at 72 °C for 15 min. The amplified fragment after purification and the empty pGBKT7 plasmid were both digested with the restriction enzymes *Nde*I and *Sal*I (NEB, Beverly, MA, USA). The total reaction mixture was 50 μl and contained 1 μg insert/plasmid, 1 μl restriction enzyme, and 5 μl 10× NEBuffer in nuclease-free water. The mixtures were incubated at 37 °C for 1 h to allow the reaction to complete. The incised insert and plasmid (pGBKT7) were purified and then ligated at 16 °C overnight with T4 DNA ligase. The recombinant pGBKT7-VirB10 plasmid was confirmed by PCR amplification and sequencing (Sangon Biotech, Shanghai, China).

**Table 1:** Primers and PCR amplification conditions

Primer name	Primer sequence (5′-3′)	Annealing temperature (°C)	Amplicon size (bp)
VB10otF	AATTC**CATATG**ATGTCAGACGAAACCAAGGATAAC	58	1326
VB10otR	TCGAC**GTCGAC**CTACCTCCGCACCGCCTC		
V10-32F	CGC**GGATCC**ATGTCAGACGAAACCAAGGATAA	58	1340
V10-32R	AGAAT**GCGGCCGC**CTACCTCCGCACCGCCTC		
S41-F	CGC**GGATCC**ATGCGCCGCCACAAGGGT	63.5	1139
S41-R	ATTT**GCGGCCGC**TCAGCGGCGGCGGATGAG		

*Note*: The bold letters indicate the inserted enzyme restriction sites

### Auto-activation and toxicity tests

The bait protein was tested for auto-activation and toxicity before the Y2H screening. Following the manufacture’s protocols for the Yeastmaker™ Yeast Transformation System 2 (Clontech, Mountain View, CA, USA), the pGBKT7-VirB10, pGBKT7, pGBKT7-53 positive control and pGBKT7-Lam negative control plasmids were transformed into Y2HGold. Transformants were grown on SD/-Trp, SD/-Trp/X (40 μg/ml X-α-Gal), SD/-Trp/X /A (40 μg/ml X-α-Gal and 125 ng/ml Aureobasidin A) or SD/-Leu/-Trp/ X-αGal/AbA (40 μg/ml X-α-Gal and 125 ng/ml Aureobasidin A) agar plates at 30 °C for 3–5 days. Only the bait plasmids that did not show auto-activation and toxicity were used in yeast two-hybrid screening.

### Mate & Plate Library screening

To screen the cDNA library for identifying potential interacting tick salivary gland proteins, the Matchmaker™ Gold Yeast Two-Hybrid System (Clontech) was used, according to the manufacturer’s protocols. Briefly, a concentrated cultured bait strain (pGBKT7-VirB10) and thawed library yeast strain (prey) were prepared and mated. The blended mixture was cultured in 2× YPDA/Kan medium and incubated at 30 °C with slow shaking (30–50× *rpm*) for 20–24 h until a typical three-lobed zygote could be seen under the microscope. The mated culture was coated on SD/-Trp, SD/-Leu and SD/-Trp/Leu medium for 3–5 days at 30 °C to determine the mating efficiency, and the remainder of the culture was grown on DDO/X/A (SD/-Trp/-Leu/X-αGal/Aba) medium for 3–5 days at 30 °C. All the blue colonies that grew on DDO/X/A were transferred onto higher stringency SD/-Ade/-His/-Leu/-Trp/X-α-Gal/AbA (QDO/X/A) agar plates for 3–5 days at 30 °C using a flat sterile toothpick.

### Selection of the positive prey plasmids

To confirm the interactions, the prey plasmids were extracted from putative positive clones using the Easy Yeast Plasmid Isolation Kit (Cat. No. 630467; Clontech). Subsequently, each prey plasmid was transformed into *Escherichia coli* DH5α competent cells (Transgen, Beijing, China). For transformation, 100 μl *E. coli* DH5α competent cells were gently mixed with extracted prey plasmids and kept in an ice bath for 30 min. Cells were then incubated at 42 °C for 45 s and returned to the ice bath for 2 min. SOC medium (500 μl) was pre-incubated at 37 °C and added to the cells, which were incubated by shaking (220× *rpm*) for 2 h at 37 °C. After plating 100 μl of culture onto LB/Amp agar plates and incubating overnight at 37 °C, the transformants growing on the LB/Amp agar plates were purified using the Plasmid Mini Kit I (Cat. No. D6943-02; Omega, Doraville, GA, USA). Following this, each putative positive prey plasmid from the initial screening was co-transformed into Y2HGold strain with the bait plasmid. Transformants were then spread onto QDO/X/A plates to test for interactions. Positive hits appeared as blue colonies under these conditions. To estimate each insert size of the potential positive prey plasmids, primers pGADT7-F (5′-TAA TAC GAC TCA CTA TAG GGC-3′) and pGADT7-R (5′-AGA TGG TGC ACG ATG CAC AG-3′), were used for PCR amplification.

### Cloning and expression of VirB10

To further validate the interactions between VirB10 and prey protein, the coding sequences of bait and prey plasmids were amplified and cloned into pET30a and pGEX-4T-1 plasmids, respectively. The coding sequence of VirB10 was amplified using primers V10-32F/R (Table 1) and cloned into a pGEM-T Easy Vector (Promega, Beijing, China). The recombinant plasmid pGEMT-VirB10 was digested with *BamH*I and *Not*I (NEB). The total 50 μl reaction mixture contained 1 μg insert/plasmid, 1 μl restriction enzyme, 5 μl 10× NEBuffer, and nuclease-free water. The mixtures were incubated at 37 °C for 1 h to fully react. After purification, the incised insert and plasmid were ligated at 16 °C overnight with T4 DNA ligase. Similarly, the coding sequence of the prey protein (UL36) was amplified using primers S41F/R (Table 1) and cloned into the pGEM-T Easy Vector. The recombinant plasmid pGEMT-UL36 was digested with *BamH*I and *Not*I and sub-cloned into pGEX4T-1 as described above. The recombinant His-bait plasmid was transformed and expressed in the *E. coli* strain BL21DE3 while the GST-prey plasmid was transformed and expressed in the *E. coli* strain BL21 (TransGen, Beijing, China). The final obtained clones were then inoculated in LB broth supplemented with kanamycin and cultured overnight. This overnight culture was used for further inoculation of fresh LB broth, and expression was induced with 1mM isopropyl-β-D thiogalactopyranoside (IPTG) at 0.6 OD. The expression of His-VirB10 and GST-UL36 was determined by western blot analysis.

### Purification of VirB10

The recombinant protein was purified under denaturing conditions. Briefly, the 50 ml induced bacterial culture was pelleted after centrifugation at 4000× *g* for 20 min and washed twice with 1× phosphate-buffered saline (PBS). The washed pellet was dissolved in 8 ml lysis buffer (8 M urea, 100 mM NaH_2_PO_4_, 10 mM Tris-Cl, 20 mM β-mercaptoethanol, 1% Triton X-100, 1 mM phenylmethylsulfonyl fluoride) at pH 8.0, sonicated for 12 min, and incubated for 2 h with continuous stirring at 175× *rpm* at room temperature. After incubation, the lysed cell suspension was centrifuged at 12,000× *rpm* for 25 min at room temperature, and the supernatant was collected and mixed with Ni-NTA agarose (Qiagen) at a 1:3 ratio (super-flow Ni-NTA slurry: lysate) for 1 h to allow the recombinant VirB10 His-tagged protein to bind with Ni^2+^ in the super-flow slurry. The agarose super-flow Ni-NTA slurry-lysate mix was then packed into a 15 ml column and flow-through was collected. The column was then washed with 20 ml wash buffer (8 M urea, 100 mM NaH_2_PO_4_, 10mM Tris-Cl, 1% Triton X-100, 10% glycerol, pH 6.3) to remove the unbound or weakly-bound non-specific proteins. The recombinant VirB10 protein was then eluted from the column using 1 ml elution buffer (8 M urea, 100 Mm NaH_2_PO_4_, 10 mM Tris-Cl, pH 4.5) for 5 fractions and further analyzed on 12% SDS-PAGE. Refolding or dialysis of the purified protein was performed with a decreasing urea gradient (8 M, 6 M, 4 M, 2 M and 1 M) and finally against 1× PBS. The concentration of purified recombinant protein was estimated by the Bradford method using bovine serum albumin (BSA) as standard [27].

### Glutathione S-transferase (GST) pull-down

Following protocols of the Pierce^TM^ GST Protein Interaction Pull-Down Kit (Thermo Fisher Scientific, Waltham, MA, USA), we equilibrated glutathione agarose. Briefly, the GST-UL36 protein was immobilized on the Pierce spin column. The His-VirB10 protein was captured by the Pierce spin column containing the immobilized GST-tagged prey protein. Then the bait-prey protein was eluted with glutathione elution buffer. To confirm that the His-VirB10 and GST-UL36 recombinant proteins did not bind to the GST and His tag, the GST-UL36 protein and GST tag were immobilized on the Pierce spin columns to capture His tag and His-VIRB10 proteins, respectively, and eluted by glutathione elution buffer. The protein elution was analyzed by western blotting with anti-His tag (1/2000, Sigma-Aldrich, Cleveland, OH, USA) or anti-GST tag mouse monoclonal antibodies (1/2000, Signalway Antibody, College Park, MD, USA). This experiment was repeated to confirm the results are credible.

### Western blot

Equivalent amounts of eluted proteins were run on a 12% SDS-PAGE gel and then transferred to PVDF membranes (Millipore, Burlington, MA, USA) using a semi-dry Trans-Blot Turbo Transfer System (BioRad, Irvine CA, USA). The membrane was incubated with a blocking buffer (TBS containing 5% BSA and 0.05% Tween-20) for 1 h at room temperature. Incubations with the diluted primary antibody in TBS 0.05% Tween-20 were performed at 4 °C overnight. After 2 h incubation with an anti-rabbit or anti-mouse peroxidase-conjugated antibody (1/5000, Sigma-Aldrich) at room temperature, the membrane was washed five times (5 min each) with PBST. The membrane was then treated with ImmunoCruz™ Western Blotting Luminol Reagent (Santa Cruz Biotechnology, Heidelberg, Germany) according to the manufacturer’s instructions.

### Sequence analysis

The sequences of the positive prey plasmids were analyzed and searched against the NCBI databases to analyze the function of the corresponding gene. The protein function of the identified gene was analyzed using Gene Ontology (http://amigo.geneontology.org/amigo), UniProt database (http://www.uniprot.org/), SMART (http://smart.embl-heidelberg.de/) and STRING (http://string.embl-heidelberg.de/).

## Results

### Construction of a yeast two-hybrid cDNA library

**Fig. 1:**
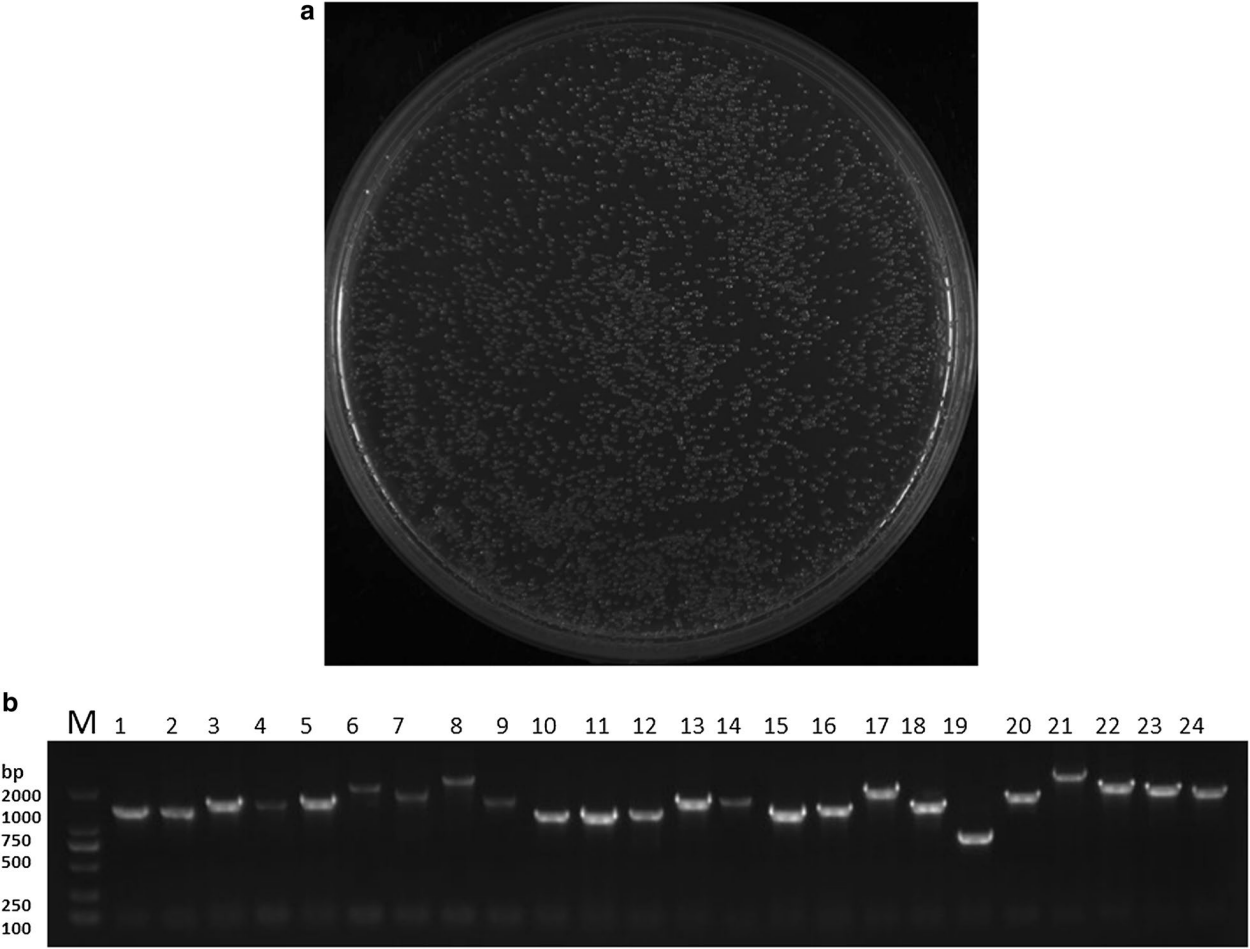
**a** Clone counts. For the dilution factor 10^−2^, the number of clones on the plate was 1200 giving 4.8 × 10^6^ cfu. **b** Determining prey inserts sizes. Agarose gel electrophoresis of amplified PCR products from 24 random clones of cDNA fragments from *D. silvarum* with average size > 1000 bp (Lanes 1–24)

The quality of the cDNA library showed the cDNA library titer was 4.8 × 10^6^ cfu (Fig. 1a), and the average size of inserted fragments was > 1000 bp, according to the PCR amplification results of 24 random clones (Fig. 1b). These results indicated that the cDNA library could be used for yeast two-hybrid screening.

### Construction of bait plasmid and testing its auto-activation and toxicity

**Fig. 2:**
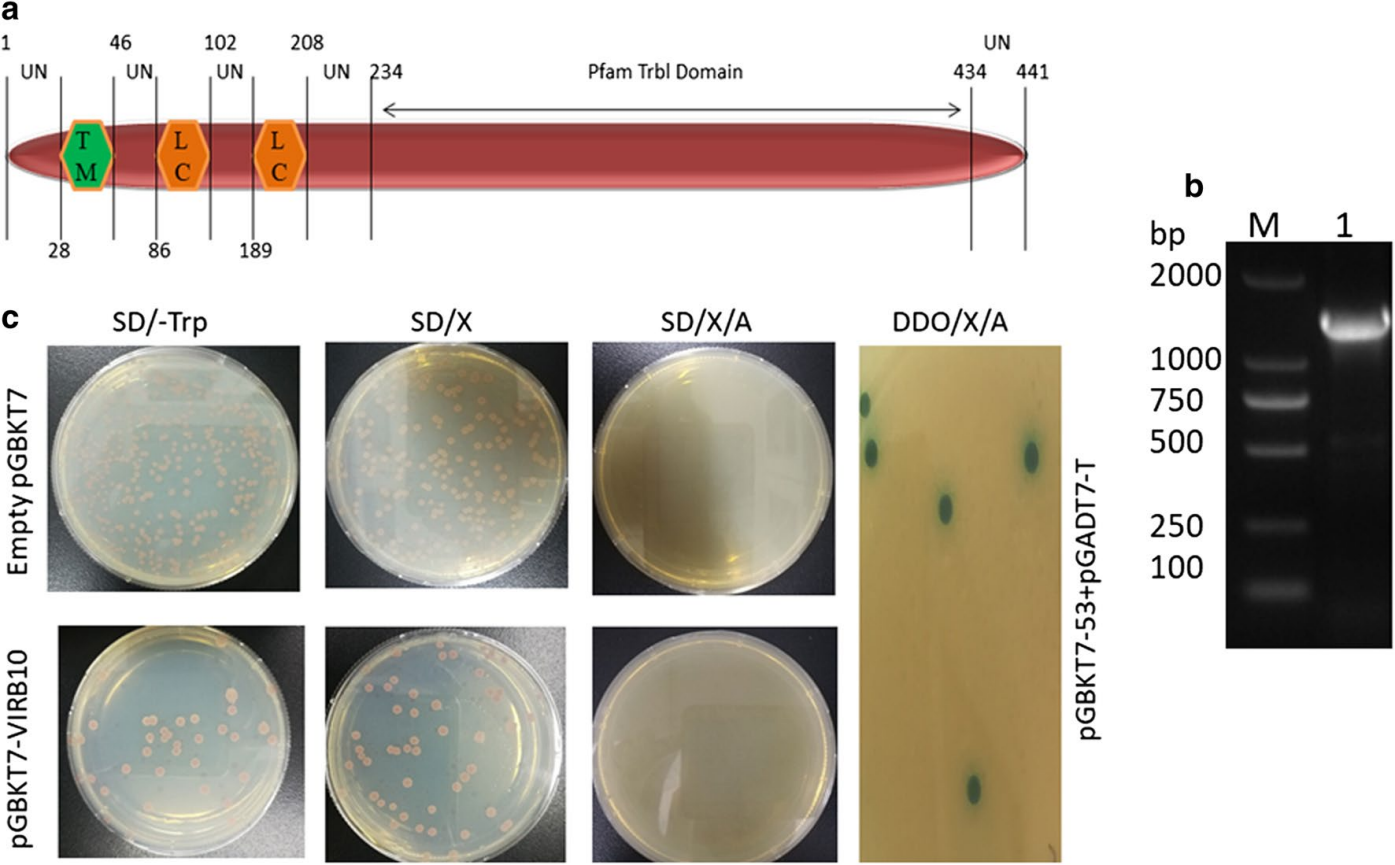
**a** Gene structure of the *virb10* bait used in yeast-two-hybrid screening. TM, LC and UN stand for the trans-membrane region, low complexity region, and unknown region, respectively. **b** Construction of pGBKT7-VirB10 bait plasmid confirmed by agarose gel electrophoresis. Lane M: DL2000 DNA marker; Lane 1: confirmation of amplified bait (pGBKT7-VirB10) clone. **c** Determination of auto-activation and toxicity for pGBKT7-VirB10 bait plasmid in yeast cells. The pGBKT7-VirB10 bait and pGBKT7 plasmids were used to transform Y2HGold cells, which were grown on separate plates. The co-transformants containing pGADT7-T and pGBKT7-53 were grown on DDO/X/A plates as a positive control

Sequence analysis of the cloned *virb10* showed it encodes 441 amino acid residues (from aa 1 to aa 441) (Fig 2a). The recombinant plasmid (VirB10-pGBKT7) was confirmed by PCR amplification (Fig. 2b). The results of the bait plasmid auto-activation testing showed that the yeast transfected with a plasmid containing pGBKT7-VirB10 or empty pGBKT7 were white on SD/-Trp/X plates, and the positive control colonies were blue on DDO/X/A plates (Fig. 2c). This proved that the recombinant plasmid pGBKT7-VirB10 did not autonomously activate the reporter genes in Y2HGold in the absence of a prey protein. The colony size of Y2HGold transformed with bait plasmid was similar to that of Y2H Gold transformed with the pGBKT7. Based on these results, the pGBKT7-VirB10 bait plasmid was qualified for use in the yeast two-hybrid screening.

### Identification of positive prey from the cDNA library

**Fig. 3:**
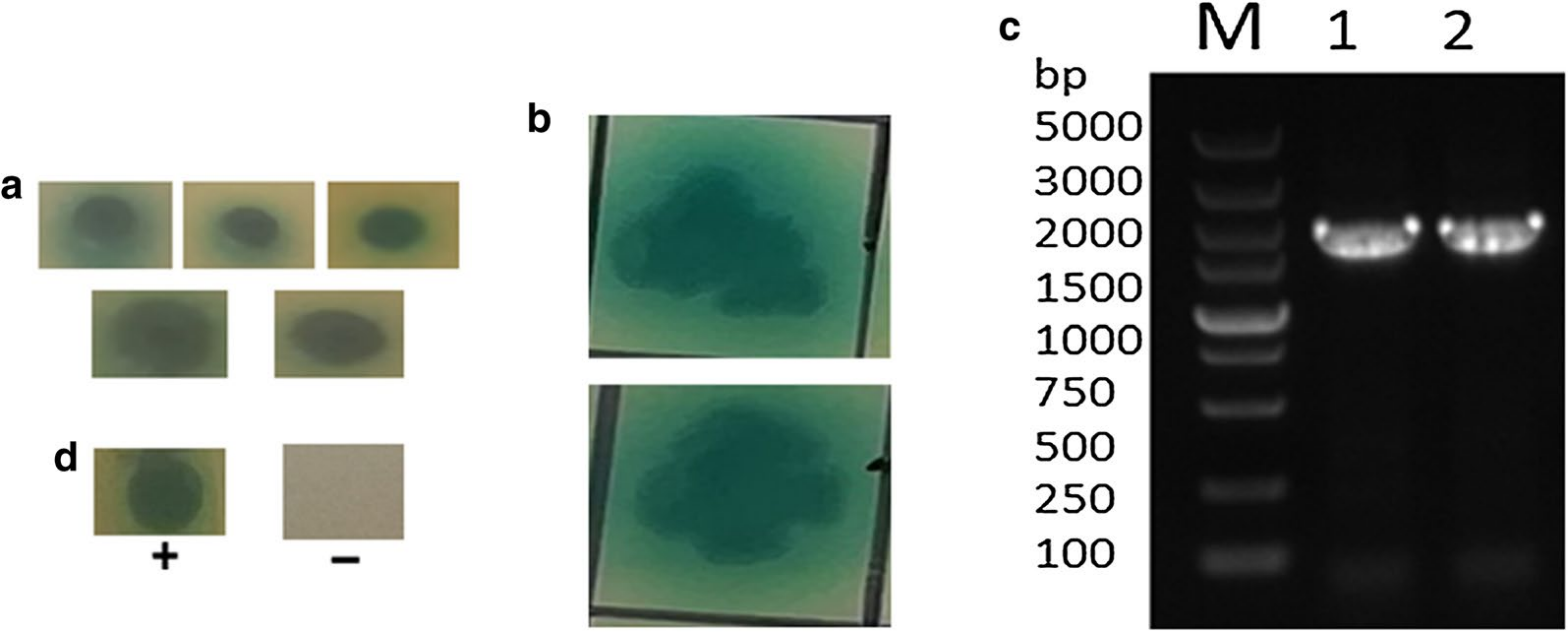
Analysis of putative positive colonies. **a** Mated bait-prey plasmids (transformed in yeast) showing blue colonies on DDO/X/A. **b** Two of the five blue clones patched out onto higher stringency QDO/X/A plates manifested blue patches. **c** Agarose gel electrophoresis of amplified PCR products for the inserts of putative positive prey plasmids. Lane M: DNA 5000 maker; Lanes 1 and 2: PCR amplification products of the inserts of the two putatively positive hits. **d** Mated prey plasmid and pGBKT7-VirB10 in Y2HGold cells cultivated on QDO/X/A plates showed blue colonies while mated prey plasmid and empty pGKBT7 showed no growth on QDO/X/A plates

After mating of bait-prey plasmids and achieving growth on the DDO/X/A plates, five blue clones were obtained (Fig. 3a). The five blue clones were subsequently re-plated twice onto higher stringency QDO/X/A plates. Two of the five colonies still manifested a blue color after two rounds on QDO/X/A, indicating that they were likely to be true positive hits (Fig. 3b). The prey plasmids were then isolated from their corresponding colonies and rescued through the transformation of *E. coli* DH5α cells. After PCR amplification using the primers pGADT7-F/R, the size of the inserted fragment in each prey plasmid was determined by gel electrophoresis (Fig. 3c). To eliminate false positive hits, both prey plasmids were mated with pGBKT7-VirB10 into Y2HGold cells and were cultivated on QDO/X/A plates, which showed blue colonies. Meanwhile, both rescued prey plasmids were mated with empty pGKBT7 plasmids into Y2HGold cells, without any growth on QDO/X/A plates (Fig. 3d).

### Sequence analysis of positive prey plasmid

To characterize the identified clones, both prey plasmids were sequenced using the primers pGADT7-F/R. The sequences were analyzed using the BLAST tool in NCBI. As a result, both plasmids showed 100% similarity with each other and 80% similarity with *Ixodes scapularis* conserved hypothetical protein-UL36 (XP_002436149.2). Gene ontology, UniProt, SMART, and STRING analyses showed the UL36 to be a large tegument protein with epidermal growth factor (EGF), which is involved in several cellular functions, including pleiotropic proliferation, developmental cell cycle control, and novel deubiquitinating activities [28–31].

### *In vitro* evaluation of the interaction between UL36 and VirB10

**Fig. 4:**
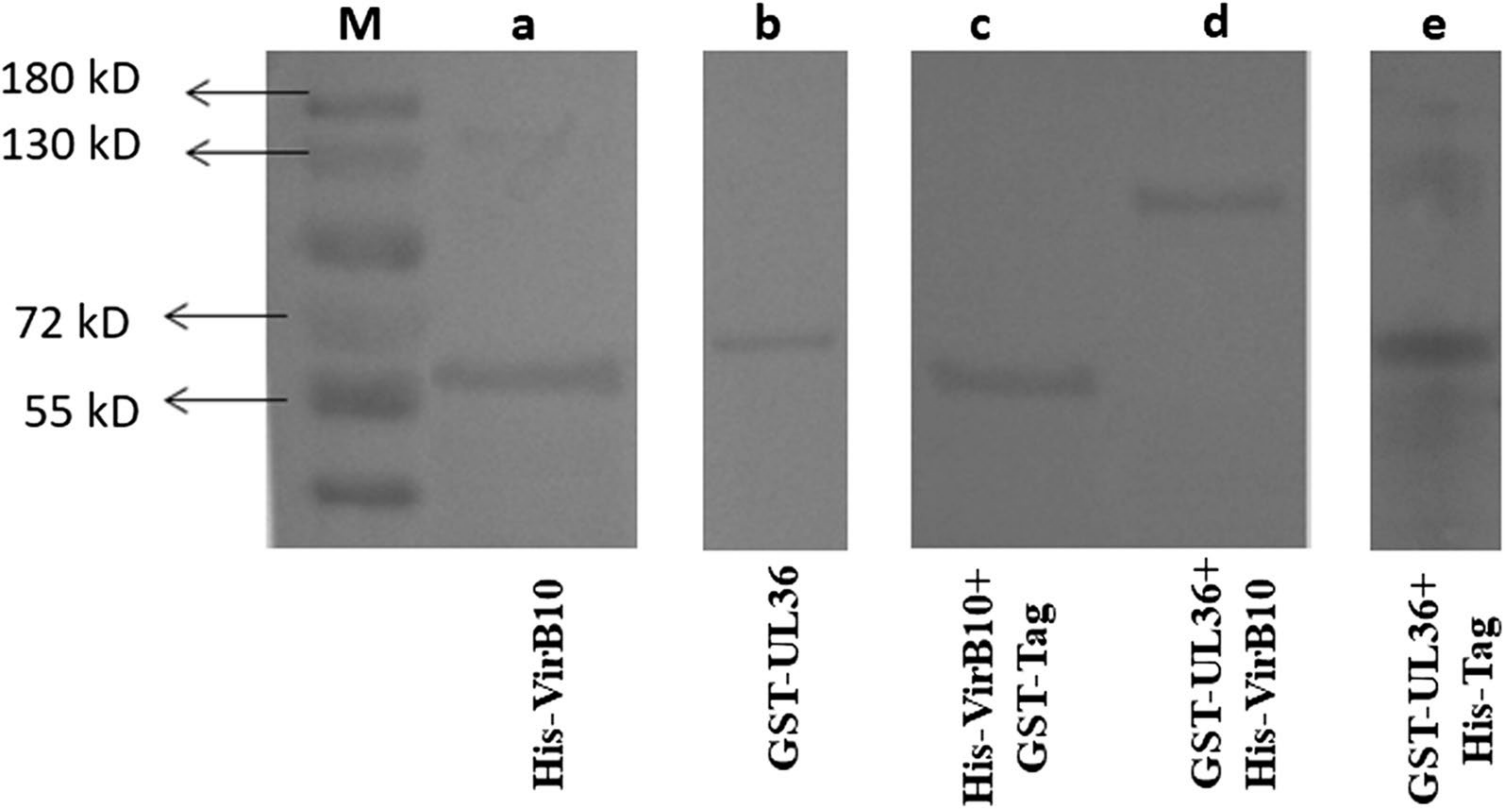
Western blots of recombinant protein expression and GST pull-down results. **a** Expression of IPTG-induced recombinant protein His-VirB10 (approximately 57.7 kD). **b** Expression of IPTG-induced recombinant protein GST-UL36 (approximately 65.5 kD). **c** The input His-VirB10 and GST-tag mixture was eluded showing no binding between the His-VirB10 recombinant protein and GST-tag protein. **d** The fusion protein GST-UL36-His-VirB10 (approximately 123 kD) was obtained after GST pull-down assay using anti-His tag. **e** The input GST-UL36 and His-tag mixture was eluded showing no binding between the GST-UL36 recombinant protein and His-tag protein

The results of western blot analysis of the recombinant protein His-VirB10, which was transformed into *E. coli* BL21 (DE3) competent cells, showed there was successful expression of a 57.7 kD protein. GST-UL36 was transformed into competent cells BL21, which successfully expressed a 65.5 kD protein (Fig. 4a, b). The recombinant proteins GST-UL36 and His-VirB10 were used for the GST pull-down assay. Eventually, the anti-His tag (1/2000, Sigma-Aldrich) was used for the western blot, and a target band of about 123 kD was obtained, proving that there was genuine interaction between the two proteins. GST pull-down showed that the GST-UL36 protein did not bind to the His-tag protein. Meanwhile, the His-VirB10 protein also showed no binding to the GST-tag protein *in vitro*. In contrast, GST-UL36 bound to His-VirB10, indicating a direct interaction between VirB10 and UL36 (Fig. 4c).

## Discussion

The immunoelectron microscopy and genetic analysis established VirB10 as a basic structural component of the T4 secretory system [16, 17]. Bacteria utilize T4SS to transfer proteins and genetic material from the bacteria to host or vector cells [32]. VirB10 is a bacterial conjugation TrbI-like protein [14] that participates in secretion, intracellular trafficking and vesicular transportation [15]. The VirB10 protein in T4SS facilitates cellular transportation by constituting a membrane channel that is incorporated into inner and outer membranes [33].

In this study, the cDNA of the *D. silvarum* salivary gland was cloned into the pGADT7-*Sma*I vector, which had three different reading frames to ensure the correct expression of each protein. Following a high-throughput yeast two-hybrid screening, two putative positive clones were obtained. However, sequence analysis showed that both clones encoded the same protein (UL36), which was found to interact with VirB10 in screening experiments, suggesting that this assay had a low false-positive rate. Sequence analysis of VirB10 using the online software SMART demonstrated that it incorporates one transmembrane region (aa 28–46) and one Tbrl domain (aa 234–434). The full *virb10* gene was cloned into the pGBKT7 vector to screen *D. silvarum* salivary gland interacting proteins using the Y2H system. Subsequently, a conserved hypothetical protein, UL36, from *D. silvarum* salivary glands was identified as interacting with VirB10. To the best of our knowledge, the present study is the first to discover that the conserved hypothetical protein, UL36, from the tick salivary gland interacts with *A. ovis* VirB10, using the Y2H and GST pull-down techniques.

UL36 has an EGF-like domain and is a large tegument protein [34, 35]. EGF is a short peptide that contains an idiosyncratic motif of six cysteines [34]. Homologous repeats of EGF catalyze proteolytic activation, which is integral to the regulation of extracellular proteolysis [36]. Various proteins with EGF domains and homologous sequences bolster the cell adhesion of normal and infected/tumor cells [37]. EGF is a protein of the coagulation and fibrinolytic pathway, suggesting these pathways are necessary for EGF interactions with receptor or ligand [31]. Several pathogenic microorganisms manipulate the ubiquitination status of host cells by both conjugation and deconjugation [38–41]. Extensive research into the Herpes virus UL36 has revealed that UL36 activity culminates at the late stages of herpes virus replication [42]. The significant cellular activities of the UL36 protein, ascertained by various studies, have included pleiotropic proliferation, developmental effects, adhesion of infected cells, and modification of ubiquitination. Although the existence of an interaction between VirB10 and UL36 is clear from the present study, the functional mechanisms involved require further investigation. Because of the peak in UL36 protein activity during the late stages of replication in the Herpes virus, we hypothesized that the VirB10-UL36 interaction plays an important role in bacteria’s virulence while in the tick salivary glands, as this is the site from where *A. ovis* is transferred to the host. Before entering the host, VirB10-UL36 might help to boost the number of infected cells; however, further functionality studies are needed to uncover the exact molecular mechanisms involved.

## Conclusions

The interaction of the *A. ovis* VirB10 and tick salivary gland UL36 protein was corroborated by the GST pull-down assay, indicating that both proteins are involved in *A. ovis* development in *D. silvarum*. Further investigations to explore the functionality of UL36 in *A. ovis* and the role of interactions between *A. ovis* and *D. silvarum* are necessary.

## Data Availability

All data generated or analyzed during this study are included in this published article.

## Abbreviations

cDNA: complementary deoxyribonucleic acid
IPTG: isopropyl β-D-1-thiogalactopyranoside
PBS: phosphate-buffered saline
PCR: polymerase chain reaction
SDS-PAGE: sodium dodecyl sulfate-polyacrylamide gel electrophoresis
TBS: tris-buffered saline
T4SS: Type IV Secretion System
Y2H: yeast two-hybrid
